# Novel use of the falciform ligament for reconstruction of the inferior vena cava and its tributary

**DOI:** 10.1016/j.jvscit.2021.05.010

**Published:** 2021-06-06

**Authors:** Janet W.C. Kung, Charing C.N. Chong, Kit-Fai Lee, John Wong, Paul B.S. Lai, Kelvin K.C. Ng

**Affiliations:** a Department of Surgery, Prince of Wales Hospital, New Territories, Hong Kong Special Administrative Region, People's Republic of China; b Department of Surgery, The Chinese University of Hong Kong, Shatin, Hong Kong Special Administrative Region, People's Republic of China

**Keywords:** Falciform, IVC, Ligament, Reconstruction, Tributaries

## Abstract

Tumor invasion into the inferior vena cava (IVC) and hepatic vein (HV) is challenging in cancer surgery with curative intent. Appropriate techniques for venous reconstruction are essential. We have described in detail a novel technique of fashioning an interposition tube graft using the falciform ligament to reconstruct the IVC and HV. The falciform ligament maintains all the benefits of an autologous tissue graft, with the added advantage of its flexibility in customizing graft dimensions. Its use in IVC and HV reconstruction has rarely been reported. The short-term outcomes with this tube graft are promising.

Various vascular reconstruction techniques for the inferior vena cava (IVC) have been described.^1^ In cases in which direct invasion or tumor thrombus involvement has occurred, resection of the IVC or hepatic vein (HV) will be inevitable for oncologic clearance. Depending on the extent of venous involvement, the IVC or HV can be repaired primarily, using a patch, or replaced using an interposition graft. Primary venorrhaphy is not always possible, and, even if technically feasible, it can cause stenosis, thrombosis, and embolism. Patch repair using autologous parietal peritoneum,^2^ pericardium,^3^ falciform ligament,^4^ or xenopericardial patch^5^ is well established. In cases with significant tissue loss of the involved vein, an interposition graft will be the only option unless established collateral vessels are present. The venous graft options include autologous veins, cryopreserved allografts, tubularized bovine pericardium, and synthetic grafts.^6^^,^^7^ For autologous grafts, donor site morbidity (ie, bleeding, vascular stenosis, end-organ damage) and the additional time required for graft harvest are the main limitations. The falciform ligament is readily available in most patients and can be harvested easily without added donor site complications during most abdominal surgeries. Although its use as a vascular substitute for portal vein reconstruction has been reported,^8^^,^^9^ its application, to the best of our knowledge, as an interposition tube graft for reconstruction of the IVC or HV has never been studied. In the present report, we have described in detail the technique of fashioning the falciform ligament into a tube graft for IVC and HV reconstruction in patients with locally advanced retroperitoneal sarcoma and hepatocellular carcinoma. Both patients provided written informed consent for the report of their case.

## Surgical techniques

### IVC reconstruction

**Fig 1:**
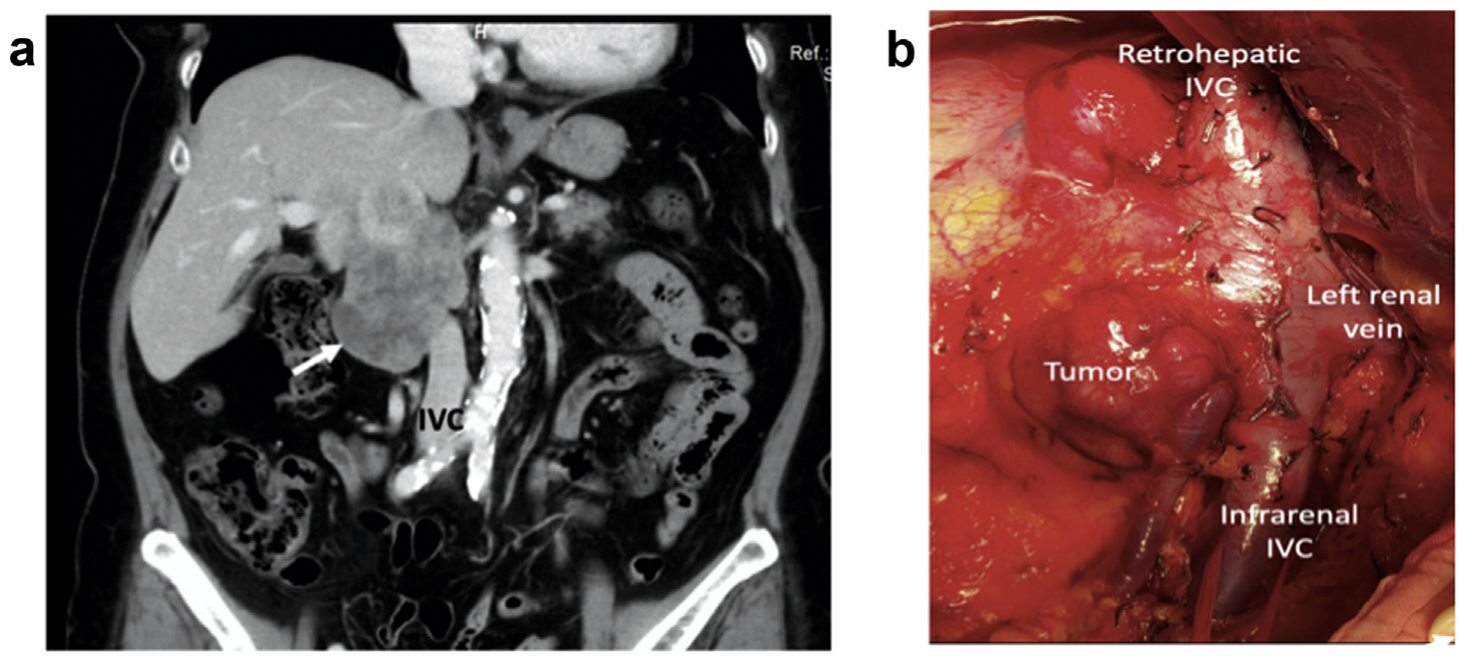
**a,** Large (12-cm) retroperitoneal leiomyosarcoma (*arrow*) with inferior vena cava (*IVC*) invasion. **b,** Vascular control of retrohepatic and infrarenal IVC and left renal vein.

**Fig 2:**
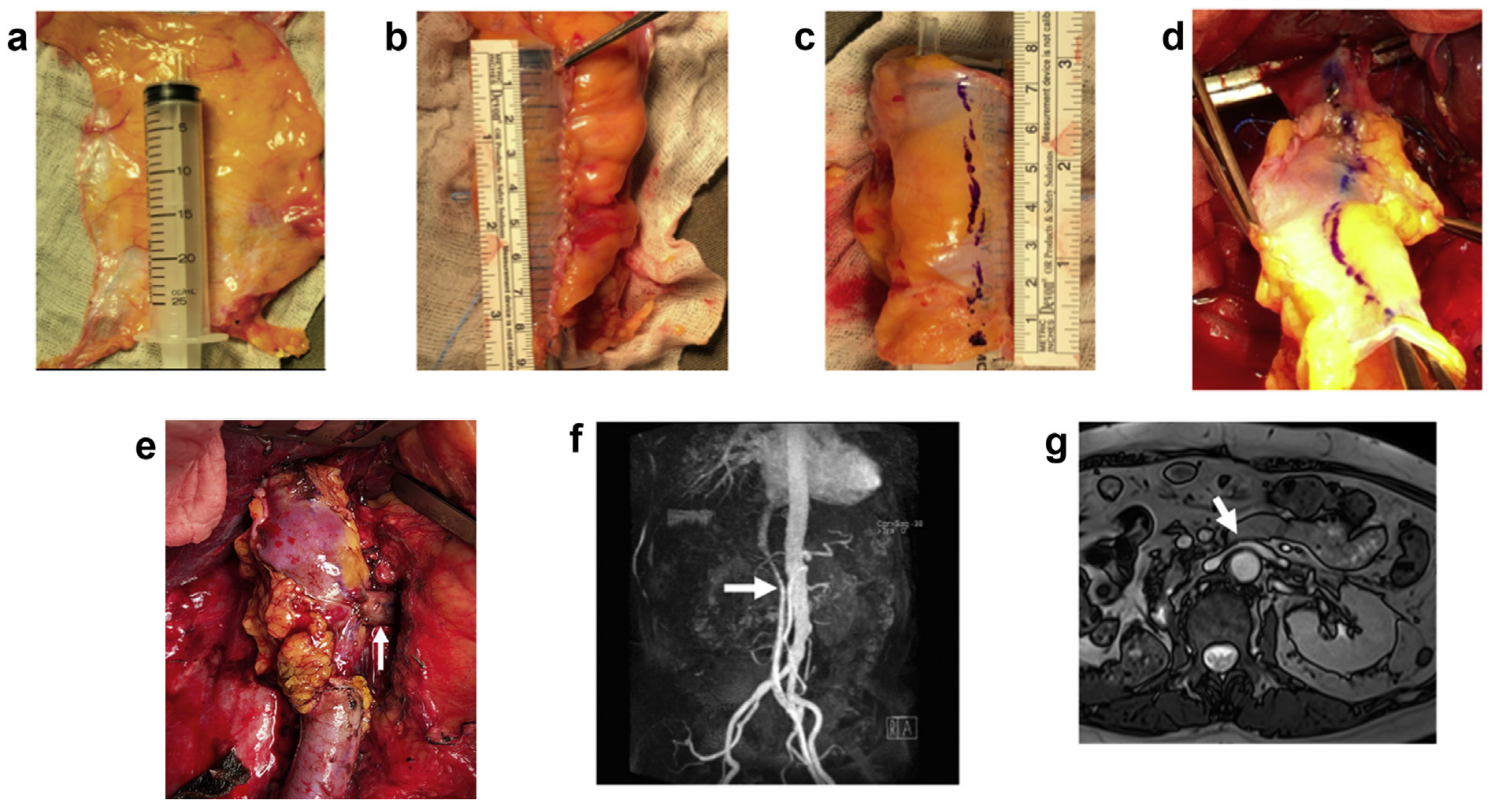
**a,** Wrapping of falciform ligament around a 20-mL syringe. **b,** The edges of the falciform ligament were sutured using 4-0 Prolene suture, and excess adipose tissue was trimmed off. **c,** Marking of the longitudinal alignment line of tube graft. **d,** Anastomoses of the interposition tube graft to the inferior vena cava (IVC). **e,** Implantation of the left renal vein (*arrow*) onto the interposition tube graft. **f,** Postoperative magnetic resonance imaging scan at 12 months showing patent falciform ligament tube graft (*arrow*). **g,** Patent left renal vein (*arrow*) on 12-month postoperative magnetic resonance imaging scan.

A 55-year-old woman had presented with a 12-cm retroperitoneal leiomyosarcoma with direct invasion into the IVC extending from the retrohepatic portion to the infrarenal portion (Fig 1, *a*). The tumor was inseparable from the right kidney and right adrenal gland. The surgical plan was en bloc tumor resection with right nephrectomy and adrenalectomy, and IVC resection with reconstruction. After laparotomy, the Cattell-Braasch maneuver was performed, exposing the IVC, aorta, and left renal vein. The infrarenal IVC was isolated and encircled. The right liver was fully mobilized and medially rotated, exposing the retrohepatic IVC, which was isolated and encircled. The left renal vein was then isolated and encircled (Fig 1, *b*). The right kidney and adrenal gland were mobilized en bloc with the retroperitoneal tumor, followed by ligation and division of the right ureter. The falciform ligament was harvested in its entirety by dissecting close to the anterior abdominal wall and anterior surface of the liver. The falciform ligament was wrapped around a 20-mL syringe (Fig 2, *a*). The edges of the falciform ligament were apposed using continuous 4-0 Prolene suture, and excess adipose tissue was dissected off to fashion a tube graft (Fig 2, *b*). The tube graft was tailored to the appropriate length and marked longitudinally using a skin marker to ensure alignment during reconstruction (Fig 2, *c*). The right renal artery was ligated and divided. The infrarenal IVC, retrohepatic IVC, and left renal vein were temporarily clamped, and a 7-cm segment of IVC was resected with the tumor. The IVC was reconstructed using the falciform ligament interposition tube graft with retrohepatic IVC and infrarenal IVC end-to-end anastomoses using 5-0 Prolene suture in a continuous, single layer with a 1-cm “growth factor” (Fig 2, *d*). The left renal vein was implanted onto the tube graft using 6-0 Prolene suture in a continuous, single-layer, end-to-side triangular manner with a 1-cm “growth factor” (Fig 2, *e*; Supplementary Video). The patient was discharged on postoperative day 7 without complications and was instructed to take aspirin 80 mg once daily for 6 months. Follow-up magnetic resonance imaging venography at 12 months after surgery showed a patent IVC graft (Fig 2, *f*) and left renal vein (Fig 2, *g*).

### Right HV reconstruction

**Fig 3:**
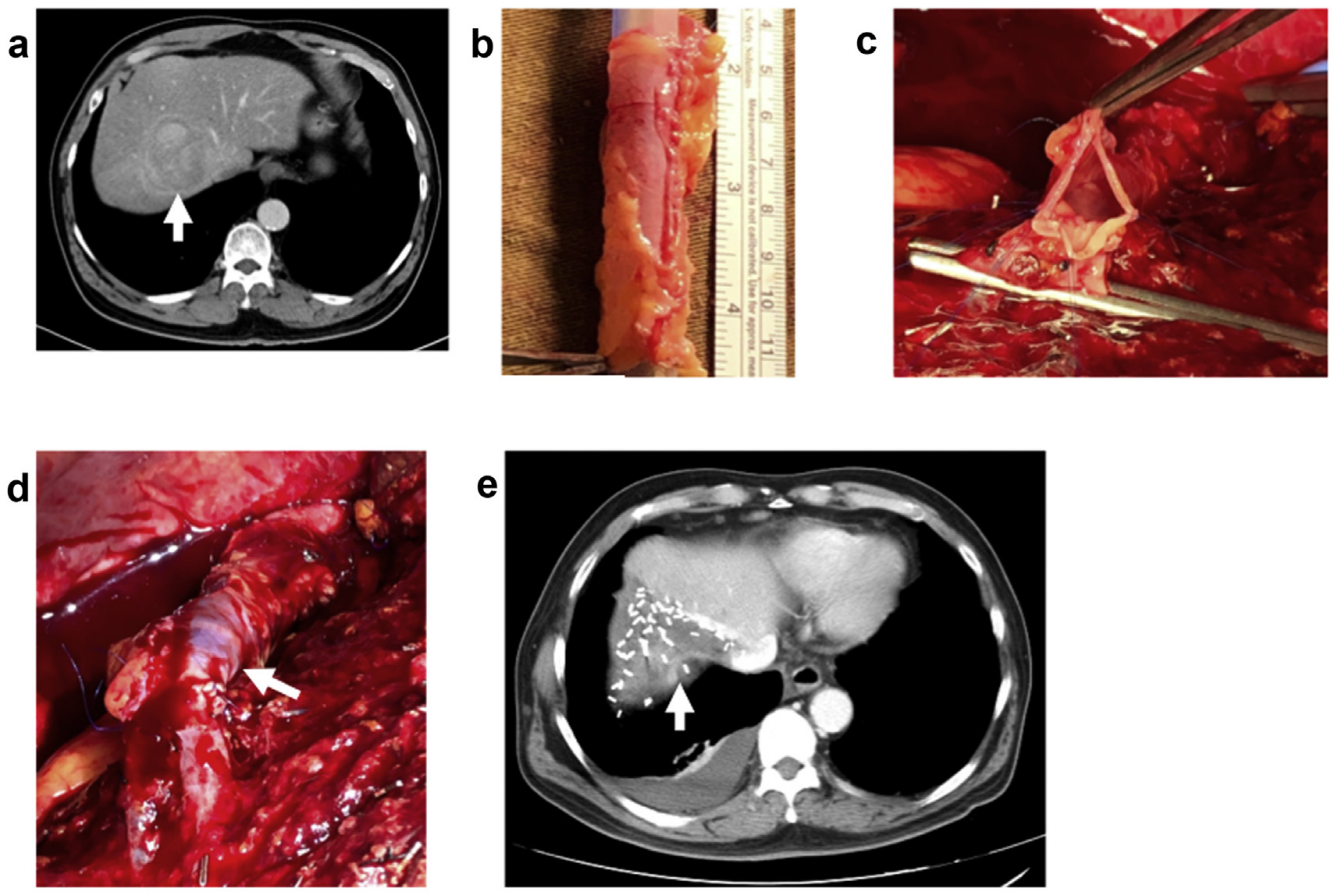
**a,** A 6-cm hepatocellular carcinoma (*arrow*) at segment VII/VIII of the liver. **b,** Fashioning the falciform ligament tube graft. **c** and **d,** Anastomoses of the interposition tube graft (*arrow*) to the right hepatic vein (HV). **e,** Postoperative computed tomography scan showing patent falciform ligament tube graft (*arrow*).

A 61-year-old hepatitis B carrier had presented with a 6-cm hepatocellular carcinoma at segment VII/VIII with direct tumor invasion into the right HV (Fig 3, *a*). The surgical plan was right superior segmentectomy (segment VII/VIII) and right HV resection with reconstruction. After laparotomy, the falciform ligament was harvested in a manner similar to that used for our first patient. The right and left triangular ligaments of the liver were mobilized to the level of the IVC and the origins of the right HV, and the common trunk of the middle and left HV insertion were isolated and encircled for outflow control. The hepatoduodenal ligament was encircled for inflow control. The hepatic transection line was determined using intraoperative ultrasound. Parenchymal transection was performed using a cavitron ultrasonic surgical aspirator with intermittent Pringle maneuvers as required. Dissection was performed to isolate the right HV at the cephalad and caudal margins of the tumor. The proximal and distal ends of the right HV were temporarily clamped, and the tumor was resected en bloc with a 6-cm segment of right HV. The falciform ligament tube graft was fashioned using a method similar to that for the previous case. The tube graft was wrapped around a 28F silicone tube, and the edges of the falciform ligament were apposed using continuous 6-0 Prolene suture (Fig 3, *b*). End-to-end venous anastomoses with the interposition tube graft were performed using continuous 6-0 Prolene suture with a 1-cm “growth factor” (Fig 3, *c* and *d*). Intraoperative Doppler ultrasound was used to confirm patency of the right HV. The patient was discharged on postoperative day 10 without complications and was instructed to take aspirin 80 mg once daily for 6 months. A follow-up computed tomography scan at 7 weeks after surgery showed a patent reconstructed right HV (Fig 3, *e*).

## Discussion

In the present report, we have illustrated the technique of using the falciform ligament as an autologous interposition tube graft for IVC and HV reconstruction. Although polytetrafluoroethylene is the standard graft material for IVC reconstruction with excellent long-term results,^10^, ^11^, ^12^ we have shown that the falciform ligament tube graft is a viable option with encouraging short-term graft patency. The use of the falciform ligament for portal venous conduits,^8^^,^^9^ hernia reconstruction,^13^, ^14^, ^15^ and peptic ulcer repair^16^ is well established; however, its application in IVC and HV reconstruction has not, to the best of our knowledge, been previously described. We acknowledge that we have only presented the short-term results, and long-term follow-up is necessary to evaluate the ultimate outcomes. The difference in caliber observed between the reconstructed IVC and native IVC (Fig 2, *f*) suggests a degree of graft compression; hence, both patients were prescribed antiplatelet therapy to promote graft patency and reduce the risk of graft thrombosis. It is anticipated that the establishment of an adequate, albeit imperfect, venous conduit would allow collateral vessels to develop over time. An increased caseload and experience would undoubtedly lead to an improved technique and better graft size assessment. Nonetheless, the benefits of using the falciform ligament are multifold. First, harvesting the falciform ligament is easy to perform and obviates the requirement for a separate incision for autologous vein harvest and the associated donor site morbidities. Second, the falciform ligament is readily available in most patients and provides a high degree of flexibility, facilitating custom tube grafts to be tailored for the individual patient. This is an expedient option, especially when the need for reconstruction is unexpected and other types of synthetic grafts are not available immediately. Third, because the falciform ligament is entirely parietal peritoneum, no “sidedness” exists with the constructed tube graft, further supporting its ease of use. Fourth, the use of this autograft reduces the risks of infection and thrombosis. Finally, its cost-effective availability has been well demonstrated.^8^

## Conclusions

The reconstruction of the IVC and HV in complex hepatobiliary and abdominal surgery using the falciform ligament as an interposition tube graft is feasible and effective. The short-term outcomes in terms of graft patency are encouraging. However, the long-term outcomes must be established.

## Appendix

Supplementary Video Anastomotic technique.
